# Studies on immunopathological changes induced by commercial IBD live vaccines in poultry birds

**DOI:** 10.1038/s41598-023-39017-5

**Published:** 2023-07-31

**Authors:** Sushma Kajal, Gulshan Narang, Babu Lal Jangir, Pooja Kundu, Deepika Lather, Rajesh Chhabra

**Affiliations:** 1 Department of Veterinary Pathology, College of Veterinary Sciences, Lala Lajpat Rai University of Veterinary and Animal Sciences, Hisar, Haryana 125004 India; 2 Department of Veterinary Public Health and Epidemiology, College of Veterinary Sciences, Lala Lajpat Rai University of Veterinary and Animal Sciences, Hisar, Haryana 125004 India; 3 College Central Laboratory, College of Veterinary Sciences, Lala Lajpat Rai University of Veterinary and Animal Sciences, Hisar, Haryana 125004 India

**Keywords:** Cytokines, Vaccines, Diseases

## Abstract

Intermediate plus live strain infectious bursal disease virus (IBDV) vaccines are used to control IBDV endemic infections in India. In the present study, immunopathological changes induced by commercial infectious bursal disease live vaccines with different immunization regimes were compared. A total of days old 108 Cobb broiler chicks were randomly divided into five groups with 24 chicks each in groups I, II, III and 18 chicks each in group IV and V. Group I served as control I (no immunization) and group II and III chicks were immunized with a single dose of vaccines 1 and 2 on 17th day of age (DOA), respectively. The group IV and V chicks were immunized with vaccines 1 and 2, respectively with primary dose on 17th DOA followed by booster dose on 24th DOA. Both intermediate plus live vaccines produced gross and histopathological lesions in lymphoid organs (bursa of Fabricius, thymus, spleen and caecal tonsils). Increased CD4 + , CD8 + T cells in affected bursa of Fabricius was evidenced by immunohistochemistry. Further, up-regulation in relative mRNA expression of IFN-γ, IL-1β and IL-6 were observed in bursa of Fabricius of treated birds, with maximum alteration particularly on 14th day post single immunization and 7th day post booster immunization. The findings suggest that single immunization regime on the 17th day of age showed immunization equivalent to booster immunization with lesser lesions, therefore, may be practiced and promoted in the field conditions for the better economic returns and animal welfare.

## Introduction

Infectious bursal disease (IBD) is an immunosuppressive and highly contagious infection of young chicken. It ranks high among the several viral diseases that cause direct financial losses to farmers^1^. In India, IBD was first reported in Uttar Pradesh and later reported from different regions of the country^2–4^. Infectious bursal disease virus (IBDV) is the causative agent of IBD, which belongs to pathogenic serotype 1 and is classified as double stranded RNA *Avibirnavirus* under *Birnaviridae* family^5,6^. The IBD is characterized by the destruction of the bursa of Fabricius (BF)^7^. The extensive IBDV replication in the BF showed alteration in B and T cells subsets which contribute to viral pathogenesis^8^. In IBDV infection, T cells enhance the viral lesions by producing inflammatory cytokines, such as IL-1β, IL-6 and IFN-γ^9,10^. The cytokines are important regulators of host immune responses and change in expression has been noted in IBDV infection^11,12^. However, scant information is available on alteration in cytokines expression following administration of IBD live vaccines by different immunization schedule in poultry birds.

Along with biosecurity measures, the use of vaccination is effective strategy for IBD control^13^. Commonly used vaccines against IBD were of Intermediate (I), intermediate plus (I+), Georgia (G), MB strains. The live strain (intermediate and intermediate plus) vaccines are extensively used to control endemic infection of IBD in India^14^. Moreover, use of intermediate plus strains of IBD virus at different attenuation levels in the commercial live strain vaccines led to varying levels of immunosuppression thereby increasing the susceptibility of birds to various concurrent infections^15^. Therefore, the safety information of these live strain vaccines still remains a major area of research.

A variety of field studies have been conducted to compare the pathological effects of different virulent strains of viruses in broiler chicken, however little is known about pathological changes and T cells in the bursal tissue following commonly used live IBD vaccines in India. The present study was hypothesized with an objective to compare immunopathological changes induced by commercial infectious bursal disease live strain vaccines with different immunization regimes in experimental broiler chicks.

## Materials and methods

The experimental study was carried out after the approval of the Institute Animal Ethics Committee (VPHE/IAEC/1702-33).

### Experimental birds

A total of day old 108-Cobb broiler chicks were procured from the local commercial hatchery (Haryana, India). The chicks were housed in the experimental house following strict hygienic condition in accordance to standard guidelines^16^. Additionally, the ARRIVE guidelines were also followed when conducting this investigation. The birds were fed on standard broiler feed and fresh water was provided ad libitum. Age of vaccination (17th and 24th days of age) was decided based on previous work on birds procured from the same hatchery^17^.

### IBD vaccines

Both commercially available intermediate plus live strain IBD vaccines 1 and 2 were procured from repute pharmaceuticals in India in the present study.

### Immunization trial

A total of day old 108-Cobb broiler chicks were randomly divided into five groups. The groups I, II, and III were comprised of 24 chicks each. The group I served as control (no immunization) and group II and III chicks were immunized with single dose of vaccines 1 and 2 on 17th day of age (DOA). The groups IV and V were comprised of 18 chicks each and immunized with vaccines 1 and 2, respectively with primary dose on 17th DOA followed by the booster dose on 24th DOA. The detailed account of experimental design is depicted in Table 1. Both vaccines were administered by intra-ocular route. The control group (I) received only vaccine diluents by intra-ocular route. Both vaccines were administered as per the manufacturer’s instruction at recommended dose.

**Table 1:** Immunization regime in the experimental birds immunized with infectious bursal diseases vaccines.

Group	Number of birds	Vaccines*	
		Primary dose^a^	Booster dose^b^
Group I (Control, no vaccine)	24	Vaccine diluent	Vaccine diluent
Group II (Single dose of vaccine 1)	24	**+**	**–**
Group III (Single dose of vaccine 2)	24	**+**	**–**
Group IV (Booster dose of vaccine 1)	18	**+**	**+**
Group V (Booster dose of vaccine 2)	18	**+**	**+**

*Two types of commercially available intermediate plus live strain vaccines were used; ^a^Single dose of vaccines were given at 17th day of age; ^b^Primary dose was given at 17th and booster dose at 24th day of age; Six birds from each group were humanely sacrificed at sequential intervals (7th, 14th, 21st and 28th day post immunization).

### Gross and histopathological studies

Six birds from each group were humanely sacrificed at sequential intervals (7th, 14th, 21st and 28th day post immunization (PI). Lymphoid organs such as BF, thymus, spleen, and caecal tonsils were examined in situ as well after removal for the gross changes. The gross lesion score (GLS) was assigned. The tissue samples from above mentioned organs were fixed in 10% neutral buffered formalin for histopathological examination. After fixation, these were processed as per the conventional procedure and tissues were embedded in molten paraffin. The sections of 4 µm thickness were cut by semi automated rotary microtome (YSI062, Yorco) and stained with haematoxylin and eosin^18^. Tissue sections were subjected for detailed histopathological examination and histopathological lesion score (HLS) was evaluated using a scale of 0–4 (0: No lesion; 1: Mild lesions (congestion and mild haemorrhages); 2: Moderate lesions (depletion of lymphocytes); 3: Moderately severe lesions (moderate depletion with necrosis); and 4: Severe lesions (severe necrosis and cysts formation) with minor modification^18^.

### Immunohistochemistry (IHC)

For IHC, BF (six from each group) were collected at weekly intervals and fixed in 4% paraformaldehyde. After fixation, similar to the histopathology, 4 µm thick sections were cut and mounted on 3-Aminopropyl-triethoxysilane coated slides. IHC was performed for the expression of CD4+ and CD8+ T cells as per the standard procedure^19^. Antigen retrieval was performed by microwave oven irradiation (800 watts) in 0.01 M citric buffer (pH 6.0) solution for 30 min (min). Blocking of endogenous peroxidase activity was done with 3% hydrogen peroxide in absolute methanol for 15 min. Before application of the primary antibodies, non-specific immunoglobulins were blocked by incubating the sections for 30 min in 5% goat serum (Sigma, Adrich). The slides were incubated overnight in the humidified chamber at 4 °C with monoclonal anti-chicken CD4 (clone CT-4; 0.5 mg/ml; 1:2) and CD8a (clone CT-8; 0.5 mg/ml; 1:10) antibodies (Southern Biotech, USA). Brick red or brown red colour cytoplasmic/membranous immunoreactivity in bursal follicles was considered as positive for both CD4+ and CD8+ T cells. The scoring was done with the following scale: + : few lightly stained positive cells; ++: widely scattered positive stained cells; +++: numerous dark stained cells in 5 randomly selected microscopic fields 400 × magnification^20^.

### RNA extraction and cDNA synthesis

Total RNA was extracted from bursal tissue collected from the experimental birds of different groups at weekly intervals (six chicks from each group) by using RNeasy Mini Kit (Invitrogen, USA) according to the manufacturer’s protocol. The purity and concentration of the extracted RNA was checked on 260/280 nm by ScanDrop^2^ (Analytik Jena). Subsequently, 500 ng purified RNA was reverse transcribed to cDNA using Revertaid™ First Strand cDNA Synthesis Kit (Thermo Scientific, USA) as per the manufacturer’s protocol.

### Quantitative Real time PCR (qPCR)

The mRNA expression of IFN-γ, IL-1β and IL-6 cytokine were analyzed by qPCR using QuantiTect SYBR Green PCR Kit (Invitrogen, USA) in cDNA samples using PikoReal Real Time PCR (Thermo Scientific, USA). Real time PCR was performed in a reaction volume of 20 μl contained 2 μl of diluted cDNA (1:10 in Nuclease free water), 10 μl of SYBR Green Master Mix, 0.5 μl each for forward and reverse primer and 7.0 μl of Nuclease free water. Primer sequences are given in Table 2^21–23^. Briefly, each reaction for IFN-γ and IL-1β involved first cycle at 95 °C for 15 min, followed by 40 cycles each of 95 °C for 15 s, 60 °C for 30 s and a final step of 76 °C for 30 s. Each reaction for IL-6 involved first cycle at 95 °C for 10 min, followed by 45 cycles each of 95 °C for 10 s, 60 °C for 10 s and a final step at 72 °C for 10 s. Subsequently, melting curve analysis was performed to check the specific product. Each sample was run in triplicate and β actin act as housekeeping gene.

**Table 2:** Primers used in quantitative real time PCR.

Target gene	Primer sequence (5′–3′)
β-Actin^21^	F: CAACACAGTGCTGTCTGGTGGTA R: ATCGTACTCCTGCTTGCTGATCC
IFN-γ^22^	F: GCCTCCAGCTCCTTCAGAATACG R: CTGGATCTGGTTGAGGAGGCTGT
IL-1β^21^	F: GTGAGGCTCAACATTGCGCTGTA R: TGTCCAGGCGGTAGAAGATGAAG
IL-6^23^	F: CACGATCCGGCAGATGGT R: TGGGCGGCCGAGTCT

### Statistical analysis

The data for all the parameters were analyzed using one-way analysis of variance (ANOVA) with Tukey’s post hoc Duncan test. The mRNA expression of IFN-γ, IL-1β and IL-6 cytokine was calculated relative to the β-actin gene and n-fold increase relative to the control samples expressed as 2^−ΔΔCt24^. Among experimental groups, pair-wise comparison was done by using SPSSTM 20.0 version and significant difference was considered at p ≤ 0.05.

## Results

### Gross and histopathological studies

The details of gross and histopathological lesion score are depicted in Tables 3 and 4. The lymphoid organs of unimmunized control birds showed normal histological architecture with no pathological changes at any time interval. Conversely, immunized birds showed gross alterations such as congestion, haemorrhages and oedema in BF. The other lymphoid organs viz., thymus, spleen and caecal tonsils also revealed congestion and haemorrhages. On 7th day PI, BF, thymus and spleen showed considerable alteration in the immunized birds (groups II and III) as depicted by significant higher (p ≤ 0.05) GLS in comparison to control birds (groups I). The GLS in lymphoid organs on 14th day PI revealed significant higher (p ≤ 0.05) in immunized birds as compared to control birds. On 21st day PI, birds immunized with single dose of vaccines (groups II and III) showed an improvement and did not reveal any significant gross morphological alterations in lymphoid organs. Whereas, considerable alteration were persistent in the lymphoid organs of booster immunized birds (group IV ad V) as depicted by significant high (p ≤ 0.05) GLS (Table 3) as compared to other birds (groups I, II and III). Thereafter, vaccine induced changes subsided and no gross lesions were observed in lymphoid organs of any group at the end of experiment (28th day PI).

**Table 3:** Gross lesion score (GLS) in lymphoid organs at weekly intervals in different experimental groups immunized with infectious bursal diseases vaccines.

GLS					
Organs	Days PI	7	14	21	28
Bursa of Fabricius	Group I	0.00 ± 0.00^A^	0.00 ± 0.00^A^	0.00 ± 0.00^A^	0.00 ± 0.00^A^
	Group II	0.83 ± 0.10^B^	1.00 ± 0.00^B^	0.00 ± 0.00^A^	0.00 ± 0.00^A^
	Group III	1.00 ± 0.00^B^	1.33 ± 0.21^B^	0.00 ± 0.00^A^	0.00 ± 0.00^A^
	Group IV	–	1.33 ± 0.21^B^	0.83 ± 0.17^B^	0.00 ± 0.00^A^
	Group V	–	1.50 ± 0.22^B^	1.00 ± 0.00^B^	0.00 ± 0.00^A^
Thymus	Group I	0.00 ± 0.00^A^	0.00 ± 0.00^A^	0.00 ± 0.00^A^	0.00 ± 0.00^A^
	Group II	0.50 ± 0.22^AB^	0.83 ± 0.17^B^	0.00 ± 0.00^A^	0.00 ± 0.00^A^
	Group III	0.67 ± 0.21^B^	1.17 ± 0.31^B^	0.00 ± 0.00^A^	0.00 ± 0.00^A^
	Group IV	–	1.17 ± 0.31^B^	0.67 ± 0.21^B^	0.00 ± 0.00^A^
	Group V	–	1.5 ± 0.22^B^	0.83 ± 0.17^B^	0.00 ± 0.00^A^
Spleen	Group I	0.00 ± 0.00^A^	0.00 ± 0.00^A^	0.00 ± 0.00^A^	0.00 ± 0.00^A^
	Group II	0.67 ± 0.21^B^	1.00 ± 0.00^B^	0.00 ± 0.00^A^	0.00 ± 0.00^A^
	Group III	0.67 ± 0.21^B^	1.33 ± 0.21^B^	0.00 ± 0.00^A^	0.00 ± 0.00^A^
	Group IV	–	1.17 ± 0.31^B^	0.67 ± 0.21^B^	0.00 ± 0.00^A^
	Group V	–	1.50 ± 0.22^B^	0.89 ± 0.17^B^	0.00 ± 0.00^A^
Caecal tonsils	Group I	0.00 ± 0.00^A^	0.00 ± 0.00^A^	0.00 ± 0.00^A^	0.00 ± 0.00^A^
	Group II	0.33 ± 0.21^A^	0.83 ± 0.17^B^	0.00 ± 0.00^A^	0.00 ± 0.00^A^
	Group III	0.50 ± 0.22^A^	1.00 ± 0.21^B^	0.00 ± 0.00^A^	0.00 ± 0.00^A^
	Group IV	–	1.17 ± 0.37^B^	0.67 ± 0.21^B^	0.00 ± 0.00^A^
	Group V	–	1.33 ± 0.21^B^	0.67 ± 0.21^B^	0.00 ± 0.00^A^

Two types of commercially available intermediate plus live strain vaccines were used; Single dose of vaccines were given at 17th day of age (0 Day PI); Primary dose was given at 17th and booster dose at 24th day of age (7 Days PI). Abbreviation: PI, post immunization; Group I (Control, no vaccine); Group II (Single dose of vaccine 1); Group III (Single dose of vaccine 2); Group IV (Booster dose of vaccine 1); Group V (Booster dose of vaccine 2); Six birds from each group were humanely sacrificed at sequential intervals (7th, 14th, 21st and 28th day PI); Among experimental groups, pair-wise comparison was done and different superscript (A,B,C) showed significant difference at p ≤ 0.05.

**Table 4:** Hisatopathological lesion score (HLS) in lymphoid organs at weekly intervals in different experimental groups immunized with infectious bursal diseases vaccines.

HLS					
Organs	Days PI	7	14	21	28
Bursa of fabricius	Group I	0.00 ± 0.00^A^	0.00 ± 0.00^A^	0.00 ± 0.00^A^	0.00 ± 0.00^A^
	Group II	0.67 ± 0.21^B^	0.83 ± 0.42^B^	1.17 ± 0.21^B^	0.83 ± 0.40^B^
	Group III	1.17 ± 0.31^B^	1.50 ± 0.34^B^	1.33 ± 0.31^BC^	0.83 ± 0.31^B^
	Group IV	–	1.67 ± 0.33^B^	2.00 ± 0.36^CD^	1.33 ± 0.21^B^
	Group V	–	2.33 ± 0.21^B^	2.17 ± 0.31^D^	1.33 ± 0.21^B^
Thymus	Group I	0.00 ± 0.00^A^	0.00 ± 0.00^A^	0.00 ± 0.00^A^	0.00 ± 0.00^A^
	Group II	0.83 ± 0.16^B^	1.17 ± 0.31^B^	0.67 ± 0.21^AB^	0.00 ± 0.00^A^
	Group III	0.83 ± 0.16^B^	1.50 ± 0.50^B^	0.83 ± 0.41^AB^	0.00 ± 0.00^A^
	Group IV	–	1.50 ± 0.42^B^	1.00 ± 0.26^B^	0.33 ± 0.21^AB^
	Group V	–	2.00 ± 0.52^B^	1.00 ± 0.36^B^	0.50 ± 0.22^B^
Spleen	Group I	0.00 ± 0.00^A^	0.00 ± 0.00^A^	0.00 ± 0.00^A^	0.00 ± 0.00^A^
	Group II	0.67 ± 0.21^B^	1.50 ± 0.22^B^	0.5 ± 0.22^B^	0.00 ± 0.00^A^
	Group III	0.83 ± 0.16^B^	1.50 ± 0.42^B^	1.00 ± 0.36^B^	0.00 ± 0.00^A^
	Group IV	–	1.67 ± 0.33^B^	1.00 ± 0.26^B^	0.33 ± 0.21^AB^
	Group V	–	1.83 ± 0.48^B^	1.00 ± 0.36^B^	0.67 ± 0.21^B^
Caecal tonsils	Group I	0.00 ± 0.00^A^	0.00 ± 0.00^A^	0.00 ± 0.00^A^	0.00 ± 0.00^A^
	Group II	0.67 ± 0.21^B^	1.00 ± 0.36^B^	0.67 ± 0.21^B^	0.00 ± 0.00^A^
	Group III	0.67 ± 0.21^B^	1.17 ± 0.31^B^	0.67 ± 0.00^BC^	0.00 ± 0.00^A^
	Group IV	–	1.67 ± 0.33^BC^	0.83 ± 0.33^C^	0.33 ± 0.21^BC^
	Group V	–	2.00 ± 0.36^C^	1.00 ± 0.31^C^	0.50 ± 0.22^C^

Two types of commercially available intermediate plus live strain vaccines were used; Single dose of vaccines were given at 17th day of age (0 Day PI); Primary dose was given at 17th and booster dose at 24th day of age (7 Days PI). *PI* post immunization; Group I (Control, no vaccine); Group II (Single dose of vaccine 1); Group III (Single dose of vaccine 2); Group IV (Booster dose of vaccine 1); Group V (Booster dose of vaccine 2); Six birds from each group were humanely sacrificed at sequential intervals (7th, 14th, 21st and 28th day PI); Among experimental groups, pair-wise comparison was done and different superscript (A,B,C) showed significant difference at p ≤ 0.05.

**Figure 1:**
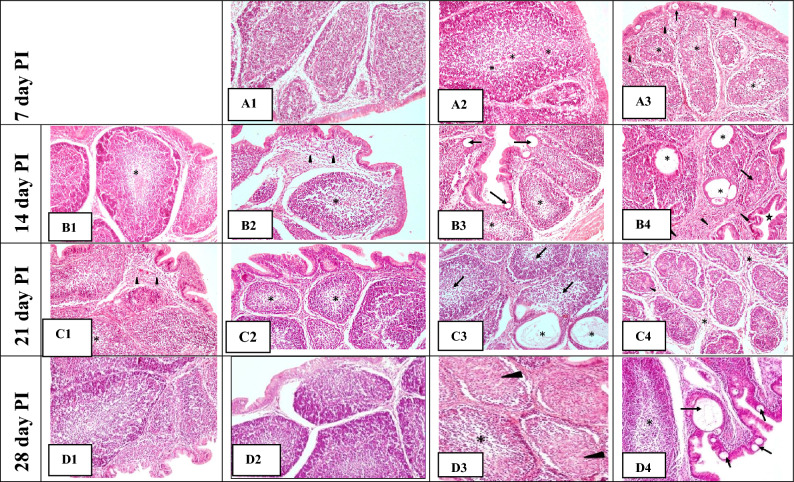
Bursa of Fabricius; (**A**) 7th day PI; A1: control group I showing normal bursal follicles. A2: Single vaccine 1 (group II) showing mild depletion of lymphocytes in medulla (asterisks). A3: Single vaccine 2 (group III) showing mild haemorrhages (arrow heads), moderate depletion of lymphocytes (asterisks) in medulla and small sized cysts in epithelium (arrows); (**B**) 14th day PI; B1: Single vaccine 1 (group I) showing depletion of lymphocytes in medulla (asterisks). B2: Single vaccine 2 (group III) showing depletion of lymphocytes in medulla (asterisk) and mild fibrous tissue proliferation (arrow heads). B3: Booster vaccine 1 (group IV) showing moderate depletion of lymphocytes in medulla (asterisks) and formation of small variable sized cysts in epithelium (arrows). B4: Booster vaccine 2 (group V) showing large variable sized medullary cysts (asterisks), atrophied follicle (arrow), tubular transformation (star) and interfollicular fibrous connective tissue proliferation (arrow heads); (**C**) 21st day PI; C1: Group II showing mild depletion of lymphocytes in medulla (asterisk) and proliferation of interfollicular fibrous tissue (arrow heads). C2: Group III showing depletion of lymphocytes in medulla (asterisks). C3: Group IV showing depletion of lymphocytes in medulla (arrows) and formation of large cystic cavities (asterisks). C4: Group V showing necrosis of lymphocytes in follicles (arrow heads), interfollicular fibrous tissue proliferation (asterisks) and atrophy of bursal follicles; (**D**) 28th day PI; D1: Group II showing almost normal appearance of histological structure. D2: Group III showing almost normal appearance of histological structure. D3: Group IV showing mild depletion of lymphocytes in medulla (asterisks) and lymphocytic necrosis (arrow heads). D4: Group V showing mild depletion of lymphocytes in medulla (asterisk) and variable sized cysts (arrows). HandE × 200.

Histopathologically, the immunized birds revealed necrosis and depletion of lymphocytes in medulla, variable sized medullary cysts, atrophied follicles and tubular transformation in BF (Fig. 1A–D). Other lymphoid organs such as thymus, spleen and caecal tonsils showed congestion, necrosis and depletion of lymphocytes. On 7th day PI, the significant higher (p ≤ 0.05) histopathological changes were observed in the immunized birds as compared to control birds. However, among immunized groups, changes were non-significant (p ≤ 0.05). On 14th day PI, BF, thymus and spleen revealed significant increase (p ≤ 0.05) in the HLS in immunized birds of all the groups as compared to control birds. However, caecal tonsils revealed significant higher (p ≤ 0.05) HLS in booster vaccine 2 (group V) birds as compared to group I, II and III. On 21st day PI, HLS of BF, spleen and caecal tonsils showed significant increase (p ≤ 0.05) in all immunized birds as compared to control birds. Thymus also revealed significant higher (p ≤ 0.05) HLS in booster immunized birds (group IV and V) only as compared to control birds. The lesions started to resolve in all the lymphoid organs thereafter, only mild lesions were observed on 28th day PI. On 28th day PI, it was observed that bursal HLS was significantly higher (p ≤ 0.05) in all the immunized groups as compared to control birds but spleen, thymus and caecal tonsils HLS were significantly more (p ≤ 0.05) in both the groups IV and V as compared to control group I (Table 4). Maximum GLS and HLS were observed on 14th day PI in booster immunized groups as compared to single immunized groups.

### Immunohistochemistry

In the present study, immunohistochemical expression of CD4+ and CD8+ T cells in bursal follicles were assessed at sequential intervals after immunization (Table 5). The IHC results revealed widely scattered reddish brown coloured immunopositive CD4+ T cells at cortico-medullary portions and diffuse infiltration of CD8+ T cells in the bursal follicles in immunized birds. Sequential study revealed that on 7th day PI, the IHC score of CD4+ and CD8+ T cells was significantly higher (p ≤ 0.05) in the groups II and III as compared to unimmunized birds (group I). On 14th day PI, CD4+ and CD8+ T cells score was significantly more (p ≤ 0.05) in all the immunized groups as compared to control group I (Table 5) and this increase was still higher in booster immunized birds (groups IV and V) as compared to single immunized birds (groups II and III). Thereafter, on 21st day PI, only CD8+ T cells score was significant higher (p ≤ 0.05) in all the immunized groups as compared to control groups (Table 5). Maximum score for both CD4+ and CD8+ T cells was observed on 14th day PI.

**Table 5:** Immunohistochemical (IHC) score in bursa of Fabricius at weekly intervals in different experimental groups immunized with infectious bursal diseases vaccines.

IHC								
Days PI	7		14		21		28	
	CD4+	CD8+	CD4+	CD8+	CD4+	CD8+	CD4+	CD8+
Group I	0.00 ± 0.00^A^	0.00 ± 0.00^A^	0.00 ± 0.00^A^	0.00 ± 0.00^A^	0.00 ± 0.00^A^	0.00 ± 0.00^A^	0.00 ± 0.00^A^	0.00 ± 0.00^A^
Group II	1.17 ± 0.31^B^	0.83 ± 0.17^B^	0.50 ± 0.22^B^	1.67 ± 0.21^B^	0.00 ± 0.00^A^	1.50 ± 0.22^B^	0.00 ± 0.00^A^	0.17 ± 0.17^AB^
Group III	1.17 ± 0.31^B^	1.17 ± 0.17^B^	0.67 ± 0.21^B^	1.83 ± 0.17^B^	0.00 ± 0.00^A^	1.67 ± 0.21^B^	0.00 ± 0.00^A^	0.50 ± 0.22^AB^
Group IV	–	–	1.50 ± 0.22^C^	2.50 ± 0.34^C^	0.00 ± 0.00^A^	2.00 ± 0.37^B^	0.00 ± 0.00^A^	0.67 ± 0.21^BC^
Group V	–	–	1.67 ± 0.21^C^	2.83 ± 0.17^C^	0.00 ± 0.00^A^	2.17 ± 0.4^B^	0.00 ± 0.00^A^	0.83 ± 0.17^C^

Two types of commercially available intermediate plus live strain vaccines were used; Single dose of vaccines were given at 17th day of age (0 Day PI); Primary dose was given at 17th and booster dose at 24th day of age (7 Days PI). *PI* post immunization; Group I (Control, no vaccine); Group II (Single dose of vaccine 1); Group III (Single dose of vaccine 2); Group IV (Booster dose of vaccine 1); Group V (Booster dose of vaccine 2); Six birds from each group were humanely sacrificed at sequential intervals (7th, 14th, 21st and 28th day PI); Among experimental groups, pair-wise comparison was done and different superscript (A,B,C) showed significant difference at p ≤ 0.05.

### Quantitative real time PCR

**Figure 2:**
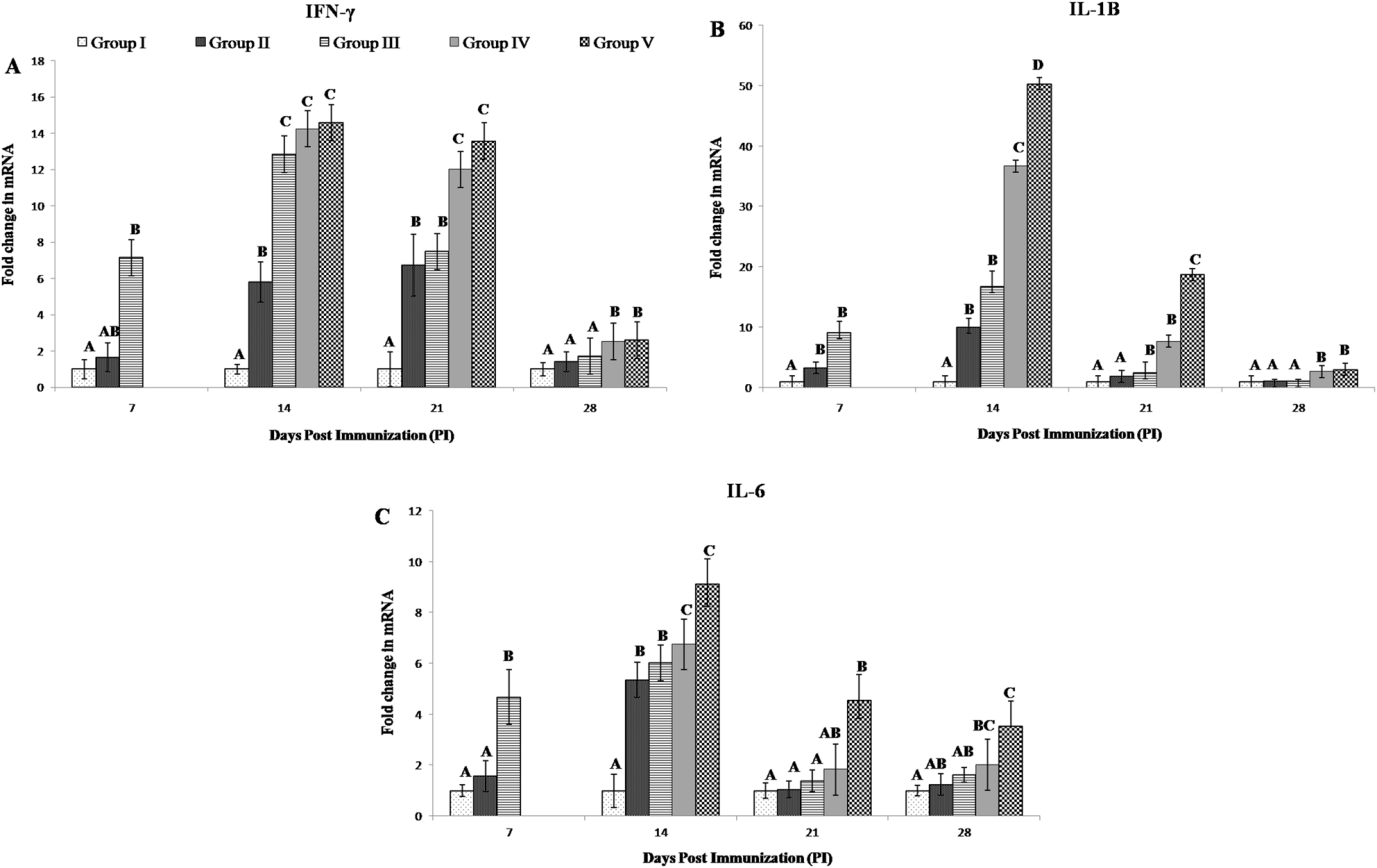
(**A**–**C**) Relative mRNA expression of IFN-γ, IL-1β and IL-6 by qPCR at different weekly intervals in different experimental groups immunized with infectious bursal diseases vaccines. Two types of commercially available intermediate plus live strain vaccines were used; Single dose of vaccines were given at 17th day of age (0 Day PI); Primary dose was given at 17th and booster dose at 24th day of age (7 Days PI). Abbreviation: PI, post immunization; Group I (Control, no vaccine); Group II (Single dose of vaccine 1); Group III (Single dose of vaccine 2); Group IV (Booster dose of vaccine 1); Group V (Booster dose of vaccine 2); Six birds from each group were humanely sacrificed at sequential intervals (7th, 14th, 21st and 28th day PI); Among experimental groups, pair-wise comparison was done and different superscript (A,B,C,D) showed significant difference at p ≤ 0.05.

The relative mRNA expression of IFN-γ was upregulated in the immunized groups as compared to unimmunized group at all intervals (Fig. 2A). On 7th day PI, maximum and significant upregulated (p ≤ 0.05) expression was found in group III as compared to control group I. On 14th PI, IFN-γ expression was found to be significantly upregulated (p ≤ 0.05) in all immunized groups as compared to control group I. Group III, IV, V also showed significant upregulation (p ≤ 0.05) as compared to group II (Fig. 2A). On 21st day PI, IFN-γ expression was found to be significantly upregulated (p ≤ 0.05) in all immunized groups as compared to control group I. Booster immunized birds (group IV and V) showed significant upregulation (p ≤ 0.05) as compared to single immunized birds (groups II and III), irrespective of vaccine (Fig. 1A). On 28th day PI, mRNA expression of IFN-γ declined in the entire immunized group and significant upregulation (p ≤ 0.05) was observed only in group IV and V as compared to group I, II and III (Fig. 2A).

The relative mRNA expression of IL-1β was found to be higher in the immunized groups as compared to unimmunize group at all intervals (Fig. 2B). On 7th day PI, relative mRNA expression for IL-1β mRNA in single immunized birds (group II and III) were significantly upregulated (p ≤ 0.05) as compared to control groups I. On 14th day PI, the expression was found to be significantly higher (p ≤ 0.05) in all immunized birds as compared to control group I. Booster immunized birds (group IV and V) showed significant upregulation (p ≤ 0.05) as compared to single immunized birds, irrespective of vaccine (Fig. 2B). Group V also showed significant upregulation (p ≤ 0.05) as compared to group IV. On 21st day PI, expression started to decline but was found to be significantly higher (p ≤ 0.05) in the immunized group III, IV and V as compared to group I and II (Fig. 2B). Group V also showed significant upregulation (p ≤ 0.05) as compared to group IV. On 28th day PI, the relative IL-1β mRNA expression in immunized birds was comparable to control group I except in booster immunized birds (group IV and V) which showed significantly higher (p ≤ 0.05) expression than other groups (Fig. 2B).

The IL-6 mRNA expression was found to be higher in the immunized groups as compared to unimmunized group during all time intervals (Fig. 2C). On 7th day PI, significant upregulated (p ≤ 0.05) expression was found in group III as compared to group I and II (Fig. 1C). On 14th day PI, the expression was found to be significantly upregulated (p ≤ 0.05) among all immunized birds as compared to control group I. Booster immunized birds (group IV and V) showed significant upregulation (p ≤ 0.05) as compared to single immunized birds, irrespective of vaccine (Fig. 2C). On 21st days PI, expression was found to be significantly higher (p ≤ 0.05) only in booster vaccinated group V as compared to group I, II and III. IL-6 mRNA expression started to decrease after that but remained significantly higher (p ≤ 0.05) in booster group IV and V) as compared to group I as observed on 28th day PI (Fig. 2C).

## Discussion

Live intermediate plus IBDV vaccines may lead to neutralization or interference with the maternal antibodies, bursal lesions and immunosuppression in chicken^15,25,26^. In the present study, immunopathological changes induced by two commercial infectious bursal disease live strain vaccines in experimental broiler chicks were compared. When, these vaccines were administered either in single dose or in booster dose at 17th and 24th DOA. In our previous work, we evaluated the immune response of these two commercially available intermediate plus IBD vaccines in day old chicks^17^. Before immunization, the ELISA mean maternal antibodies (MAbs) level were 7874.21, 2449.4 and 1089.9 in day old, 7th and 14th day of age, respectively. The mean MAbs level shows a steady decline from day old age to 14th day of age. The age of vaccination in experimental birds was determined based on MAbs level and it was confirmed to be 17th days of age. After immunization, mean antibody titres in vaccinated birds at various intervals revealed that vaccine 1 showed an increase in ELISA antibody levels, whereas vaccine 2 showed a much higher level of antibody levels starting on day 7th and both vaccines remained protective throughout the experiment. Bursal index (BI) of same chicks immunized with these two commercially available intermediate plus IBD vaccines was also calculated in our previous work and found that BI was comparable with control in case of birds vaccinated with vaccine 1 and reduced significantly in birds vaccinated with vaccine 2 at 7th day PI. Later the bursal index was significantly lower in all the vaccinated groups vaccinated with either of the vaccine at 14th, 21th, 28th day PI^27^. However, functional T cells or B cells assay would be helpful to conclude and further explore the association of bursal index and immunosuppression. In the present study, booster immunized groups (IV and V) showed higher degree of gross and histopathological lesion score in lymphoid organs particularly on 14th day PI as compared to single immunized groups (II and III). The gross findings in the present study were in agreement with other researcher who also reported similar findings such as congestion in BF, thymus, spleen and caecal tonsils in broiler chicken vaccinated with live attenuated intermediate plus IBDV vaccine^28^. Haemorrhages in visceral organs strongly suggested the damage to blood vessels possibly due to immune mediated damage caused by the effect of macrophage derived interleukins^29^. In lymphoid organs, histopathological lesions started from 7th day PI till the end of experiment (28th days PI) in all immunized birds. The severity of lesions was more evident on 14th and 21st day PI. In corroboration to our findings, other researchers have also observed mild to severe changes with vacuolation and depletion of lymphoid cell in bursal follicles^27,30–32^. Immunosuppressive nature of intermediate plus vaccine due to severe lesions in BF of vaccinated chickens have also been reported by other researchers^13,33^. The hot vaccines provide better protection but are not safe as these carry the higher inherent risk of reversion to virulence and may result in immunosuppression in chickens^5^. However, some strains of the MB vaccine may have adverse impacts on the bursa which might lead to immunosuppression^34^.

Replication of IBDV in BF induces bursal damages which indirectly affect the cellular immune responses by altered antigen presenting cells^35^. In our study, the infiltration of CD8+ T cells was more diffuse as compared to CD4+ T cells in bursal follicles on 14th day PI as evident by higher immunohistochemistry score. The previous researcher reported that intermediate plus IBDV vaccinated birds showed significantly higher levels of CD4+ and CD8+ T cells as compared to control birds at 21 days PI in BF^29^. Increase in the number of intrabursal T cells and significant reduction in B cells in bursa with moderate and severe lesions score was also previously reported in very virulent IBDV infection^36^. T cell response was higher in the birds infected with invasive intermediate strains of virus than chickens inoculated with mild (Lukert) strain, even when tenfold higher doses of inoculums were used and suggested a definite correlation between the virulence of IBDV strain and degree of T cell reaction in the BF^20^. The decrease in CD4+ and CD8+ immunoreactive positive T cells on 28th days PI as observed in present study suggested the recovery phase. The intrabursal T cells may be crucial in clearing of viruses and fostering restoration after infection^37^. The bursal CD4+ T cells may give the signals needed for B cells to alter isotypes and promoting the generation of protective antibodies. The presence of CD8+ T cells in bursal follicles with IBDV strongly suggested viral clearance by cytotoxic T lymphocytes. The cortico-medullary boundary was probably reflecting the route of entry of IBDV as it is rich in arterioles and venules, which allow the entry of lymphocytes into follicles in response to an appropriate chemo-attractants^38^. In the present study, the immunized groups showed the presence of numerous CD4+/CD8+ T cells in the bursal follicles. The previous study also reported progressive infiltration of CD4+ and CD8+ T cells in IBD infected BF from 4 day post immunization onwards and increase in the number of T cells up to 65% population of bursal cells at 7 day post immunization^37^. The bursal follicles are comprised of B cells, T cells and other non-lymphoid cells. These cells usually constitute 85–95% B cells and less than 4% T cells. In the bursal cortex and medulla after IBDV infection, there is a fast, gradual loss of B cells^37^. Further, flow cytometry would be helpful to substantiate our finding with depletion of B cells in BF. The infiltration of CD4+ and CD8+ T lymphocyte led to greater damage in the BF by producing cytokines and having a cytotoxic impact that causes persistent immunesuppression following IBD^39^.

Cytokines are small proteins, which are produced in response to infection. Chicken cytokines have been classified as pro-inflammatory IL- 1β, IL-6, IL-8 and T helper 1 (IFN-γ, IL-2, IL-8)^40^. In the present study, there was upregulation in IFN-γ, IL-1β and IL-6 mRNA expression in immunized chicks as compared to control particularly at 14th day PI. The previous researcher reported that intermediate plus vaccine group showed significantly (p < 0.05) lower levels of IFN-γ expression in birds at 12 and 24 h post-stimulation in comparison with birds in other groups (poly I:C only, Pam3CSK4 only, combination treatment and unvaccinated control)^32^. Other demonstrated that IL-18 (superfamily of IL-1β) is a pro-inflammatory cytokine which causes proliferation of CD4+ T helper cells and induce the production of IFN-γ^41^. The influx of T cells was observed at the site of viral replication and infiltrating T cells are involved in limiting viral spread in BF and initiating the recovery process but, it is also possible that T cells may enhance lesions as reported by^42^. In IBDV infection, upregulation of pro-inflammatory cytokines might be associated with immunosuppression as reported by earlier researchers^43,44^.

Both types of intermediate plus vaccine produced different degree of lesions in lymphoid organs as observed in the present study. These lesions were also evidenced by increase in CD4+, CD8+ T cells and upregulated expression of cytokine genes such as IFN-γ, IL-1β and IL-6 in the bursal tissue with maximum alteration on 14th day post single immunization (PI) and 7th day post booster immunization. The findings suggest that single immunization regime on the 17th day of age showed immunization equivalent to booster immunization with lesser lesions, therefore, it may be practiced and promoted in the field conditions for the better economic returns and animal welfare. As a result, it can be concluded that age of vaccination, different immunization regimes and immunopathological changes induced by them must be considered while formulating the dosage regimen of live vaccines.

## Data Availability

All data analysed during this study are included in this article.
